# The dual DPP4 inhibitor and GPR119 agonist HBK001 regulates glycemic control and beta cell function *ex* and *in vivo*

**DOI:** 10.1038/s41598-017-04633-5

**Published:** 2017-06-28

**Authors:** Yi Huan, Qian Jiang, Gang Li, Guoliang Bai, Tian Zhou, Shuainan Liu, Caina Li, Quan Liu, Sujuan Sun, Miaomiao Yang, Nan Guo, Xing Wang, Shusen Wang, Yaojuan Liu, Guanqiao Wang, Haihong Huang, Zhufang Shen

**Affiliations:** 1 State Key Laboratory of Bioactive Substances and Functions of Natural Medicines, Institute of Materia Medica, Chinese Academy of Medical Sciences and Peking Union Medical College, Beijing, China; 2 Organ Transplant Center, Tianjin First Center Hospital, Tianjin, China; 3 Key Laboratory for Critical Care Medicine of the Ministry of Health, Tianjin First Center Hospital, Tianjin, China

## Abstract

Glucagon like peptide-1 (GLP-1) plays a vital role in glucose homeostasis and sustaining β-cell function. Currently there are two major methods to enhance endogenous GLP-1 activity; inhibiting dipeptidyl peptidase-4 (DPP4) or activating G protein-coupled receptor 119 (GPR119). Here we describe and validate a novel dual-target compound, HBK001, which can both inhibit DPP4 and activate GPR119 *ex* and *in vivo*. We show that HBK001 can promote glucose-stimulated insulin secretion in mouse and human primary islets. A single administration of HBK001 in ICR mice can increase plasma incretins levels much more efficiently than linagliptin, a classic DPP4 inhibitor. Long-term treatment of HBK001 in KKAy mice can ameliorate hyperglycemia as well as improve glucose tolerance, while linagliptin fails to achieve such glucose-lowing effects despite inhibiting 95% of serum DPP4 activity. Moreover, HBK001 can increase first-phase insulin secretion in KKAy mice, suggesting a direct effect on islet β-cells via GPR119 activation. Furthermore, HBK001 can improve islet morphology, increase β-cell proliferation and up-regulate genes involved in improved β-cell function. Thus, we have identified, designed and synthesized a novel dual-target compound, HBK001, which represents a promising therapeutic candidate for type 2 diabetes, especially for patients who are insensitive to current DPP4 inhibitors.

## Introduction

Type 2 diabetes mellitus (T2DM) is a chronic disease characterized by insulin resistance and defective insulin secretion^1, 2^. During the development of T2DM, pancreatic β-cell function gradually declines^3^ which results in a severe deduction of insulin secretion^4^ and, ultimately, β-cell death^5^.

Glucagon like peptide-1 (GLP-1) is one of the major incretins released from intestinal enteroendocrine L cells and plays an important role in nutrient metabolism and glucose homeostasis^6^. Mechanistically, GLP-1 stimulates glucose-dependent insulin secretion without the occurrence of hypoglycemia^7^. Moreover, GLP-1 regulates β-cell proliferation and cytoprotection through multiple signal transduction pathways^8, 9^. However, the half-life of bioactive GLP-1 is less than 2 minutes due to rapid inactivation by the proteolytic enzyme, dipeptidyl peptidase-4 (DPP4)^10^. Thus, inhibiting DPP4 can slow down GLP-1 degradation and thus increase endogenous bioactive GLP-1 levels^11, 12^. In addition, GLP-1 secretion can be enhanced by activating some G-protein coupled receptors (GPRs), such as GPR119, which is highly expressed in both pancreatic β-cells^13^ and intestinal enteroendocrine L cells^14^. It is reported that GPR119 activation in L cells triggers GLP-1 release and GLP-1 receptor activation, which stimulates glucose-dependent insulin secretion (GSIS) in β-cells. Furthermore, GPR119 activation in β-cells can directly stimulate GSIS^15^.

Previous studies have shown that some diabetic individuals exhibit GLP-1 deficiency^16^ but DPP4 inhibitors fail to effectively promote islet β-cell survival in these cases^17^. Interestingly, combinatory treatment with both a DPP4 inhibitor and a GPR119 agonist was demonstrated to have benefits on alleviating hyperglycemia and improve islet β-cell regeneration^14, 18^. Recently, we proposed a new strategy of developing novel compounds which target both DPP4 and GPR119 by combining pharmacophores of GPR119 agonists and linagliptin, the classical DPP4 inhibitor^19^.

Here, we report a newly-developed compound, (R)-8-(3-aminopiperidin-1-yl)-7-(but-2-yn-1-yl)-3-methyl-1-(3-(4-(3-phenyl-1,2,4-oxadiazol-5-yl)piperazin-1-yl)propyl)-1H-purine-2,6 (3H, 7H)-dione (named HBK001 hereafter), which is digitally-designed and synthesized according to our previous work on combinatory strategies of DPP4 inhibitors and GPR119 agonists^19–21^. We hypothesize that HBK001 could both inhibit GLP-1 degradation and induce GLP-1 production by targeting DPP4 and GPR119 simultaneously *ex* and *in vivo*.

## Results

### HBK001 selectively inhibits DPP4 *ex* and *in vivo*

**Figure 1:**
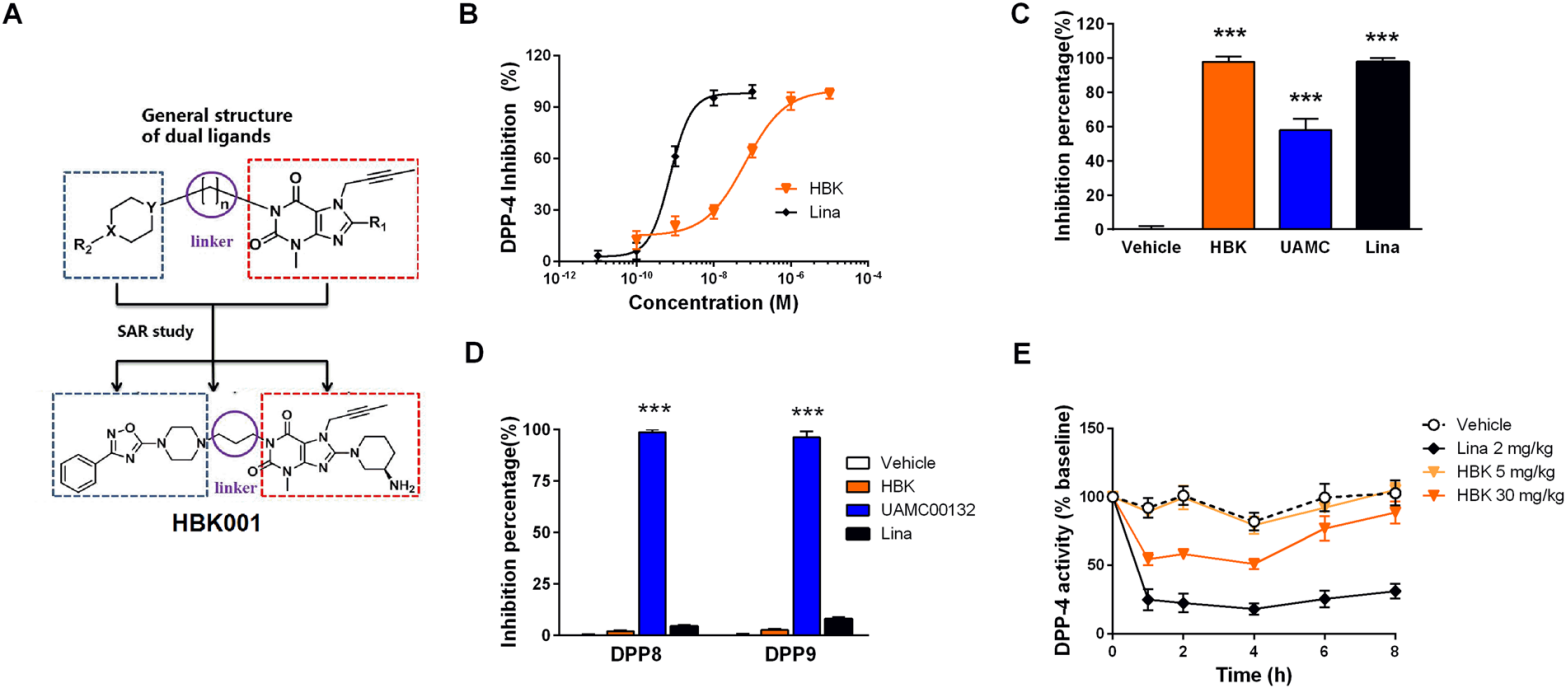
HBK001 selectively inhibits DPP4 *ex vivo* and *in vivo*. (**A**) Chemical structure of HBK001 and its pharmacophore design strategy as DPP4 inhibitor and GPR119 agonist. (**B**) Concentration-dependent inhibitory effect of HBK001 (HBK) on DPP4 activity. Linagliptin (Lina) was used as a positive control for DPP4 inhibition. (**C**) Inhibitory effect of HBK001 on purified recombinant DPP4 (0.01 μg). (**D**) Inhibitory effect of HBK001 on selectivity analysis for DPP8 and DPP9. Lysates (100 μg) from HEK293 cells transfected with the plasmid pcDNA3.1-hDPP8/9 were used in the enzymatic assay. The concentration of all compounds was 10 μM. UAMC00132 is a selective inhibitor for DPP8/9. (**E**) Serum DPP4 activity at 1 h, 2 h, 4 h, 6 h and 8 h after oral administration of HBK001 in 4 h-fasted ICR mice. Data are presented as mean ± SEM (**A** to **D**, n = 3; **E**, n = 10). ***P < 0.001 versus Vehicle.

The structure and pharmacophore-design of HBK001 was described. By targeting DPP4 and GPR119, it was designed by combining pharmacophores of GPR119 agonists and the DPP4 inhibitor, linagliptin, via a linker^19^ (Fig. 1A). To determine the effect of HBK001 on DPP4 activity, we performed the DPP4 activity assay. Our results showed that the IC50 of HBK001 and linagliptin was 66 nM and 0.75 nM respectively (Fig. 1B). HBK001 inhibited the DPP4 enzymatic activities up to 97.8% (10 μM), similar to the effect of linagliptin at the same concentration (Fig. 1C). Since the amino acid sequences of DPP8 and DPP9 are very similar to DPP4 and the fact that some studies have reported that DPP8 and DPP9 inhibition cause severe toxicities^21^, we therefore evaluated the effect of HBK001 on DPP8/9 activities. Our data suggested that HBK001 has no significant effect on either DPP8 or DPP9, while the selective DPP8/9 inhibitor, UAMC00132, inhibited DPP8/9 activity more than 96% under the same conditions (Fig. 1D). Next we used ICR mice, a rodent model with euglycemic levels, to determine DPP4 activity *in vivo* after HBK001 treatment. HBK001 inhibited serum DPP4 activity in a dose-dependent manner (Fig. 1E), and at the dose of 30 mg/kg, it achieved approximately 50% DPP4 inhibition compared to baseline for about 4 hours. Notably, 2 mg/kg linagliptin inhibited DPP4 activity more efficiently than 30 mg/kg HBK001, achieving approximately 75% inhibition efficacy. These data suggested that HBK001 selectively inhibits DPP4 enzymatic activity *ex vivo* and *in vivo* without any significant side effects via DPP8/9, but not as efficient as linagliptin.

### HBK001 activates GPR119 and induces insulin secretion *ex vivo* and incretins release *in vivo*

**Figure 2:**
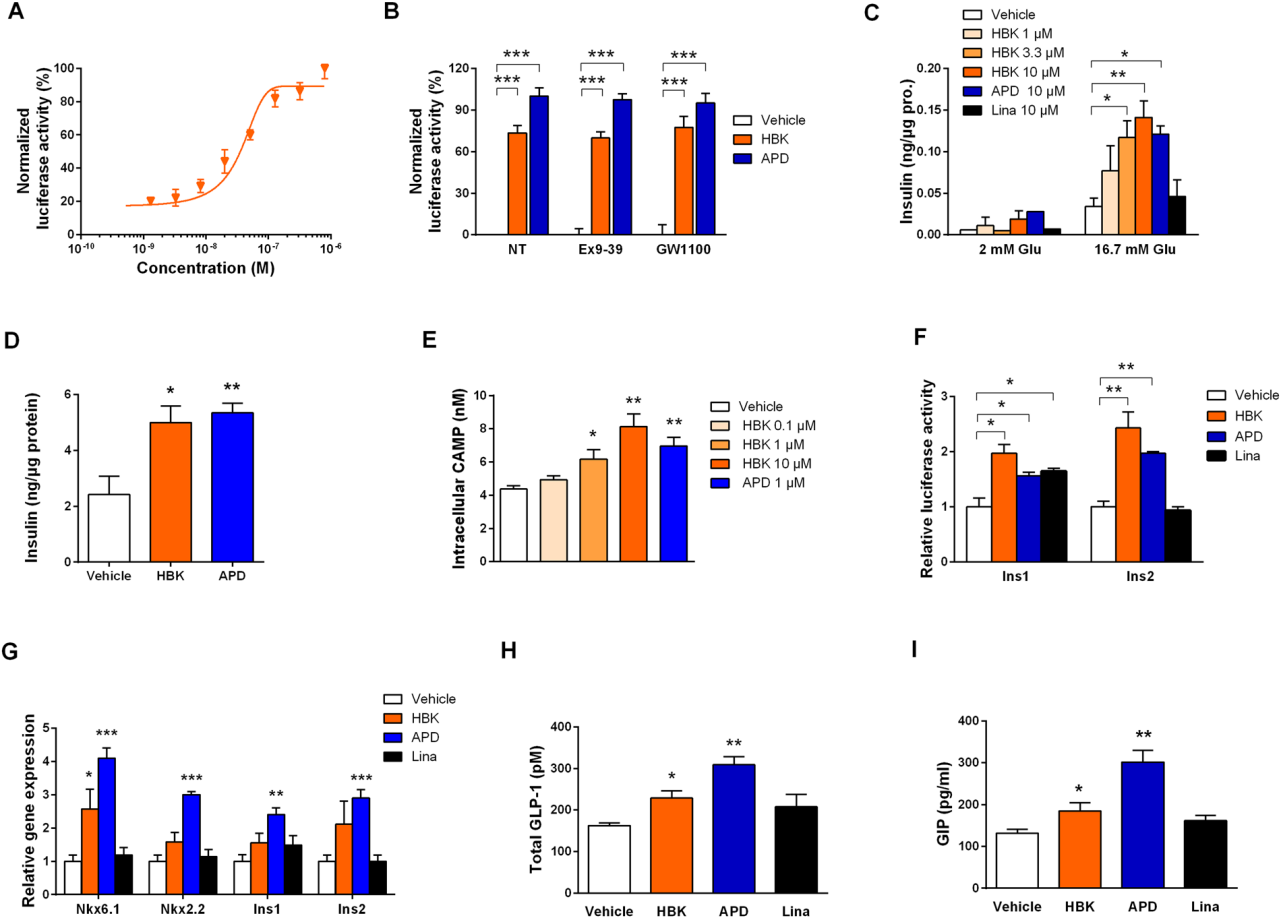
HBK001 activates GPR119 and induces insulin secretion *ex vivo* and incretins release *in vivo*. (**A**,**B**) HBK001 exhibits GPR119 activation efficacy in HEK293 cells. HEK293 cells were transiently co-transfected with Peak13-CD5L-hGPR119, pcDNA3.1-Gal4-CREB and Peak12-Gal4UAS-luci plasmids, and then treated with HBK001 at indicated concentrations for 24 hours and luciferase expression in cell lysates was measured by chemiluminescence. (**A**) Concentration-dependent activity of HBK001 on GPR119 transactivation; (**B**) Activation potency of HBK001 (10 μM) on GPR119. Data were normalized to the positive control, APD597 (Maximum activity was set as 100%). At the same time, the compounds were added with antagonists of GLP1R and GPR40, Ex9-39 and GW1100, respectively, or without these antagonists (NT) to identify whether inhibition of GLP1R or GPR40 influences the transactivation of GPR119 by the compounds. The concentration of Ex9-39 and GW1100 was 1 μM and 10 μM, respectively. (**C**) Insulin secretion in isolated primary islets from ICR mice in the presence of glucose (2 mM or 16.7 mM) and different compounds as indicated. The concentration of insulin was quantified by ELISA and was expressed as a ratio to the concentration of total protein in islets. (**D**) Insulin secretion in isolated primary islets from a human donor in the presence of 16.7 mM glucose and different compounds as indicated. The concentration of insulin was quantified by ELISA and was expressed as a ratio to the concentration of total protein in islets. (**E**) Intracellular cAMP production in mouse NIT-1 cells after treatment of compounds at the concentration of 10 μM. (**F**) Determination of HBK001 on transactivation of mouse insulin gene 1 and 2 (Ins1/2) in HEK293 cells co-transfected with Peak13-CD5L-hGPR119, Peak12-Ins1/2-luci and pRL-TK. (**G**) The effect of HBK001 on gene expression correlated with insulin secretion was explored using real-time PCR assay in mouse primary islets after 24 hours of compound treatment. In all of above experiments, solvent DMSO was used as vehicle control, HBK001 and linagliptin was treated at the concentration of 10 μM or as specifically indicated in the figure. (**H**,**I**) Serum total GLP-1 (**H**) and GIP (**I**) levels at 10 min after the glucose loading in 4 h-fasted ICR mice administered with vehicle (distilled water), HBK001 (30 mg/kg), APD597 (20 mg/kg) and linagliptin (2 mg/kg) by gavage 1 hour prior to oral glucose load (2 g/kg). Data are presented as mean ± SEM (**A** to **G**, n = 3; H, I CTGCAAAGGTTTGTCCC, n = 10), *P < 0.05, **P < 0.01, ***P < 0.001 versus Vehicle.

We determined GPR119 transactivation activity induced by HBK001 and found that HBK001 activated GPR119 ranging from 3.28 nM to 1 μM (EC50 = 0.03 μM, Fig. 2A) in a concentration-dependent manner. At a concentration of 10 μM, the ability of HBK001 to activate GPR119 reached 73.6% of APD597, a classical GPR119 agonist. Next, considering GPR40, GLP1R and GIPR are other members of the GPCR family with similar functions to GPR119 in islet β-cells^22^; we assessed the effect of HBK001 on GPR40, GLP1R and GIPR (Supplementary Fig. 1A–C). Our results indicated that HBK001 (10 μM) had no effect on these receptors. Moreover, treatment of antagonists of GLP1R and GPR40, Ex9-39 and GW1100 respectively, had no obvious influence on the transactivation activity induced by HBK001 and APD597 (Fig. 2B).

To investigate whether HBK001 has direct effects on pancreatic β-cells, insulin secretion was analyzed in isolated primary islets from ICR mice after HBK001 treatment (Fig. 2C). When treated with 16.7 mM glucose, HBK001 enhanced insulin secretion in primary islets in a concentration-dependent manner ranging from 1 μM to 10 μM. Interestingly, 3.3 μM HBK001 showed a similar ability to enhance insulin secretion as APD597 at 10 μM. However, these compounds had no effect on islets incubated with 2 mM glucose. Importantly, compared to the vehicle control group, the linagliptin-treated group did not increase insulin secretion at either 2 mM or 16.7 mM glucose. In line with this finding, HBK001 promoted GSIS in the islet β-cell line, rat insulinoma INS-1 cells (Supplementary Fig. 1D). To explore whether HBK001’s GSIS effect can be reproduced in humans, we isolated human islets from healthy donors and exposed them to HBK001, our result showed that HBK, like as APD597, could indeed promote GSIS in human islets (Fig. 2D).

GPR119 couples with Gαs^23^ which is followed by adenylate cyclase activation, protein kinase A activation and finally phosphorylation of the cAMP-response element binding (CREB) protein. Therefore, we measured the intracellular cAMP concentration in the mouse pancreatic β-cell line NIT-1 after HBK001 treatment. Consistently, HBK001 treatment increased cytosolic cAMP in a concentration-dependent manner (Fig. 2E). In addition, inhibitors of adenylate cyclase (MDL22330A) and protein kinase A (H-89), individually attenuated GSIS in islets of ICR mice treated with HBK001 (Supplementary Fig. 1E). Furthermore, we found that HBK001 and APD597 significantly increased CREB phosphorylation in NIT-1 cells at 10 μM (Supplementary Fig. 1F), suggesting that HBK001 activates the GPR119 signaling pathway similar to APD597.

To explore whether these compounds regulate insulin gene transcription in islet β-cells, a luciferase reporter gene under the control of mouse insulin gene promoter 1 (Ins1) as well as insulin gene promoter 2 (Ins2) were co-transfected into NIT-1 cells. Our results revealed that HBK001 and APD597 could significantly increase both Ins1 and Ins2 transcription compared to vehicle treatment, while linagliptin had no such effect on Ins2 (Fig. 2F). Moreover, we analysed gene expression profiles in primary islets from ICR mice treated with different compounds by real-time PCR. Our results suggested that genes involved in insulin transcription and secretion were upregulated by HBK001 (Fig. 2G).

Additionally, we evaluated glucose-stimulated incretins secretion *in vivo*, which are proposed consequences of GPR119 activation. After a single administration of HBK001 (30 mg/kg) and APD597 (20 mg/kg) in ICR mice, glucose-induced total GLP-1 levels were increased by 41.0% and 90.2%, respectively, (Fig. 2H) and glucose-induced GIP levels were increased by 40.2% and 169.9%, respectively, (Fig. 2I), while linagliptin administration (2 mg/kg) had no significant increase of either GLP-1 or GIP. The results illustrate that endogenous incretins release was promoted by HBK001 and was partially due to GPR119 activation.

Inhibition of gastric emptying is one of the biological effects of GLP-1 and GIP. To investigate whether HBK001 had an effect on gastric emptying *in vivo*, we measured blood acetaminophen levels following oral administration of acetaminophen in ICR mice which were administrated a single dose of HBK001 (30 mg/kg) and APD597 (20 mg/kg). The result showed that HBK001 could inhibit gastric emptying and resulted in a decline of blood acetaminophen levels after 30 min and 60 min of oral administration, similar to APR597 (Supplementary Fig. 2A).

### HBK001 improves glucose tolerance in ICR mice, and ameliorates hyperglycemia in diabetic db/db mice and KKAy mice

To investigate the effects of HBK001 *in vivo*, euglycemic ICR mice, as well as diabetic db/db mice and KKAy mice, were used to explore the glucose-lowing effect of HBK001, and were compared to the DPP4 inhibitor, linagliptin, as a positive control.

**Figure 3:**
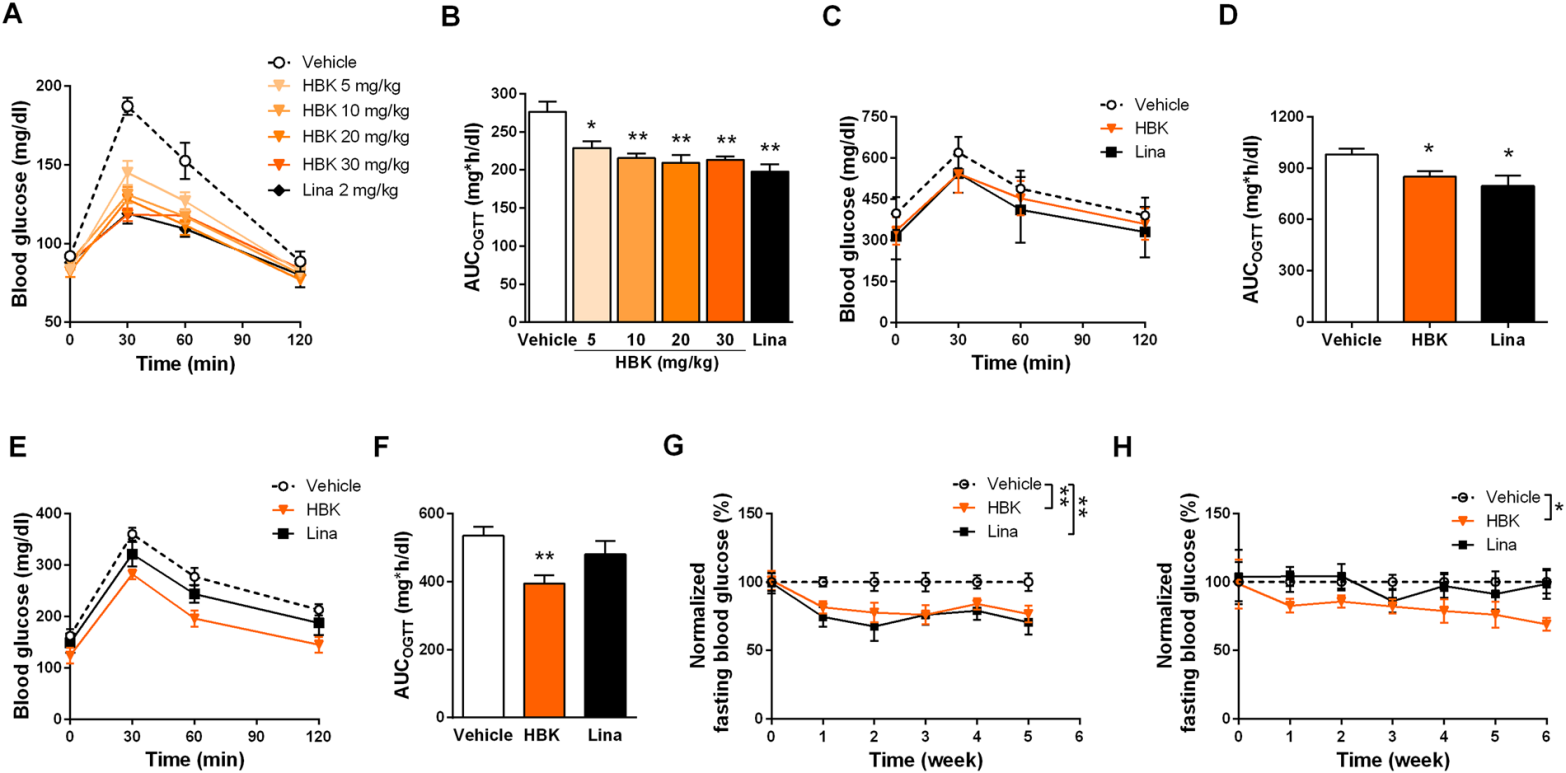
Glucose-lowering effect of HBK001 treatment in ICR mice, diabetic db/db mice and KKAy mice. (**A**,**B**) Blood glucose curves and area under the curve (AUC) of OGTT in ICR mice. (**C**,**D**) Blood glucose curves and AUC of OGTT on the 28^th^ day of HBK001 treatment in db/db mice. (**E**,**F**) Blood glucose curves and AUC of OGTT on the 33^rd^ day of HBK001 treatment in KKAy mice. To perform OGTT, overnight-fasted mice were orally administered with HBK001 (at the indicated doses) or linagliptin (2 mg/kg) 1 hour prior to oral glucose load (2 g/kg). Blood glucose levels were determined at 0, 30, 60, 120 minutes after glucose load (**G**,**H**). Fasting blood glucose levels were monitored weekly during the treatment period of HBK001 and linagliptin in db/db mice (**G**) and KKAy mice (**H**). Data are presented as mean ± SEM (n = 10), *P < 0.05, **P < 0.01 ***P < 0.001 versus Vehicle.

Oral glucose loading and tolerance test (OGTT) were utilized to assess DPP4 inhibition efficacy^24, 25^. In ICR mice, a single administration of HBK001 (5, 10, 20 and 30 mg/kg) significantly reduced blood glucose levels 30 minutes after oral glucose loading. Impressively, HBK001 (at doses 10, 20 and 30 mg/kg) could achieve a comparable effect to linagliptin (Fig. 3A). The area under the curve (AUC) of HBK001-treated groups significantly decreased by 17.1%, 21.8%, 24.2%, and 22.8% at the doses of 5, 10, 20, and 30 mg/kg respectively, and the AUC of the linagliptin-treated group decreased by 28.3% at the dose of 2 mg/kg (Fig. 3B). These findings indicated that HBK001 achieved hypoglycemic plateau at the dose of 30 mg/kg, although the glucose lowing effect of HBK001 at single administration was weaker than linagliptin.

To evaluate anti-diabetic effects of HBK001, we performed long-term oral administration with HBK001 in db/db mice and KKAy mice. Over the 28 days of treatment, the OGTT blood glucose curves of HBK001-treated (30 mg/kg) db/db mice were significantly lower than vehicle-treated mice and comparable to linaliptin treatment (2 mg/kg) (Fig. 3C). The AUC in HBK001- and linagliptin-treated groups was decreased by 13.0% and 18.6% respectively, compared to the vehicle-treated group (Fig. 3D). During the 33 days of treatment in KKAy mice, 30 mg/kg of HBK001 significantly decreased the OGTT blood glucose curves, compared to vehicle treatment (Fig. 3E). The AUC in HBK001-treated groups decreased by 26.5% compared to the vehicle-treated group. Surprisingly, linagliptin had no effect on OGTT at the dose of 2 mg/kg in KKAy mice (Fig. 3F).

Fasting blood glucose levels in diabetic mice were monitored every week during the treatments. In db/db and KKAy mice, HBK001 treatment (30 mg/kg) decreased hyperglycemic levels comparable to linagliptin treatment (2 mg/kg) (Fig. 3G and H). However, linagliptin treatment failed to normalize blood glucose in KKAy mice (Fig. 3H). Furthermore, this glucose-lowering effect of HBK001 in KKAy mice was also reinforced by significantly reduced HbA1c levels (Supplementary Fig. 2B), suggesting better glycemic control effects of HBK001 treatment compared to linagliptin.

### HBK001 promoted insulin secretion and improved islet β-cell function in KKAy mice via a GPR119-dependent pathway

**Figure 4:**
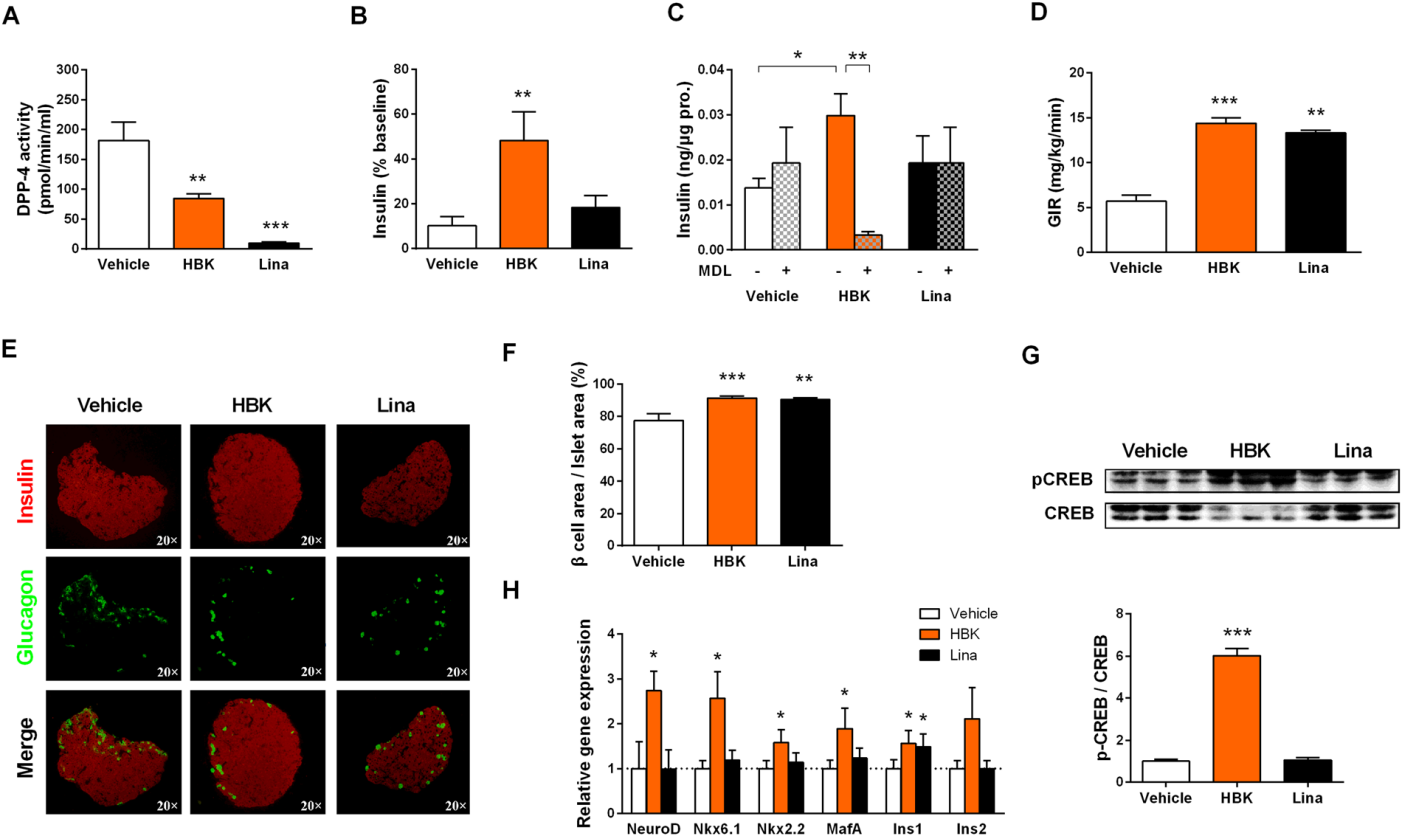
HBK001 promoted insulin secretion and improved islet β-cell function in KKAy mice via a GPR119-dependent pathway. (**A**) Blood DPP4 activity measurements on the 48^th^ day of HBK001 treatment. (**B**) HBK001 treatment increased first-phase insulin secretion in the hyperglycemic clamp test. Insulin secretion levels were measured 5 min after glucose infusion. (**C**) Glucose-stimulated insulin secretion (GSIS) determination in primary islets of KKAy mice. Primary islets isolated from KKAy mice in the presence of 16.7 mM glucose and 10 μM HBK001 or linagliptin while DMSO was used as a vehicle control. At the same time, the adenylyl cyclase inhibitor, MDL12330A (MDL), was added to block production of cyclic adenosine monophosphate (cAMP). (**D**) HBK001 treatment increased the glucose infusion rate (GIR) in the hyperglycemic clamp test. (**E**) HBK001 treatment normalized islet morphology and increased β-cell area in the pancreas of spontaneous type 2 diabetic KKAy mice. Immunostaining of insulin (red) and glucagon (green) in pancreatic sections. (**F**) β-cell percentage of total islet area. (**G**) Western blot analysis of CREB protein phosphorylation in pancreas of KKAy mice treated with HBK001 and linagliptin. (**H**) Quantitative PCR analysis of genes related to β-cell function and survival in KKAy mice treated with HBK001. A comparative cycle threshold (CT) method was used for relative quantification of gene expression between different groups; β-actin was used for normalization. Data are presented as mean ± SEM (**A**, n = 10; **B**,**D**–**F**,**H**, n = 5; **C**,**G**, n = 3), *P < 0.05, **P < 0.01, ***P < 0.001 versus Vehicle.

Linagliptin was reported as one of the strongest DPP4 inhibitors^24, 26^ and showed efficient glucose-lowering effects in diabetic db/db mice in our hands. Surprisingly, HBK001 treatment showed better glucose-lowering effects than linagliptin treatment in diabetic KKAy mice. To explore the underlying mechanism, we performed serum DPP4 activity assay in KKAy mice (Fig. 4A). HBK001 treatment (30 mg/kg) inhibited ~50% DPP4 activity, while linagliptin treatment (2 mg/kg) inhibited 95% activity of serum DPP4. Secondly, evaluation of first phase insulin secretion, which was induced by oral glucose challenge, indicated that administration of HBK001 significantly enhanced insulin secretion by 4.7-fold compared to the vehicle group, while linagliptin had no effect (Fig. 4B). We also performed *ex vivo* GSIS determination using primary islets from KKAy mice, and showed that HBK001 promoted insulin secretion in a GPR119-dependent manner which was blocked by inhibiting adenylate cyclase using the compound MDL12330A. In contrast, linagliptin had no direct effect (Fig. 4C). Importantly, there was no significant difference in fasting blood insulin levels between vehicle and HBK001-treated groups on the 48th day (Supplementary Fig. 2C), while blood glucagon levels were decreased by 13.2% (*P* = 0.065) in HBK001-treated KKAy mice (Supplementary Fig. 2D), suggesting that long-term treatment of HBK001 did not affect basal insulin levels without glucose challenge and might decrease glucagon levels.

During long-term treatment in KKAy mice, body weight and food intake were monitored every two days. The results showed that HBK001 treatment had no influence on body weight (Supplementary Fig. 2E) while food intake was slightly decreased (Supplementary Fig. 2F) and possibly caused by inhibition of gastric emptying.

In addition, we also explored serum GLP-1 levels in KKAy mice. Unfortunately, GLP-1 levels in KKAy mice were undetectable when using normal ELISA methods. However, 2 mg/kg of linagliptin treatment in KKAy mice for 48 days failed to decrease hyperglycemia although it could sufficiently block DPP4 activity. This result suggested that DPP4 inhibition was not suitable for treatment in a diabetic animal model which had deficiency in endogenous GLP-1 production. Moreover, this also suggested that the improved glucose-lowering effect of HBK001 in KKAy mice compared to linagliptin was due to GPR119 activation and direct effects on islet β-cells.

On the last day of the treatment, hyperglycemic clamp testing was performed to assess islet β-cell function (Fig. 4D). HBK001 treatment (30 mg/kg) significantly increased the glucose infusion rate, indicating the maximum capacity of β-cell insulin secretion was improved, which was substantially greater than linagliptin treatment (2 mg/kg).

To determine whether HBK001 had any effect on the morphology of islets or the β-cell area percentage, islets were double-stained for insulin and glucagon (Fig. 4E). Islets in normal mice were comprised of a large insulin-positive β-cell core surrounded by glucagon-positive α-cells punctate on the surface. However, in vehicle-treated diabetic KKAy mice, the morphology of the islets was remarkably changed and the glucagon positive α-cells were significantly increased. Intriguingly, treatment with HBK001 dramatically normalized islet morphology, and the ratio of insulin-positive β-cell/total islet area was increased by 17.9% after 48 days of treatment (Fig. 4F). Moreover, using immunostaining of PCNA to mark cell proliferation in pancreatic sections from KKAy mice, we demonstrated that long-term HBK001 treatment increased PCNA staining in islets of KKAy mice which indicates that HBK001 may induce regeneration of islet cells (Supplementary Fig. 2G).

As long-term HBK001 treatment significantly increased β-cell area in KKAy mice, we next questioned whether the GPR119 signaling pathway and gene expression profiles involved in β-cell function and survival were activated by HBK001. Western blot analysis in the pancreas of KKAy mice showed that long-term treatment with HBK001 increased CREB phosphorylation, indicative of GPR119 pathway activation (Fig. 4G). Using real-time PCR, we found that several key genes regulating β-cell function and survival, such as NeuroD, Nkx6.1, Nkx2.2, MafA, and insulin genes, were significantly up-regulated in HBK001-treated mice compared to the vehicle-treated control mice (Fig. 4H), reinforcing the idea that HBK001 had positive and beneficial effects on β-cell function and survival.

## Discussion

Progressive dysfunction of pancreatic β-cells and decline in β-cell mass are major characteristic features of T2DM and there is an increasing demand for drugs and therapies to ameliorate pancreatic dysfunction^1, 15, 27^. GLP-1 is an incretin hormone which can stimulate β-cell proliferation and enhance glucose-dependent insulin secretion and thereby improve pancreatic function^28^. Numerous studies seeking new approaches to increase endogenous GLP-1 levels have been reported for potential T2DM treatment. Notably, either GPR119 agonists or DPP4 inhibitors, can enhance GLP-1 levels by stimulating its release or by preventing its degradation, respectively. Ansarullah *et al*. reported that a combination of a GPR119 agonist (PSN632408) and a DPP4 inhibitor (sitagliptin) could significantly increase active GLP-1 levels and stimulated β-cell regeneration^14^. However, to our knowledge, there are no reports so far for a dual-targeted compound with active pharmacophores for both DPP4 inhibition and GPR119 activation.

Here we report that HBK001, a novel small-molecule compound synthesized by linking part of linagliptin to the GPR119 pharmacophore, can both enhance GPR119 and inhibit DPP4 activities simultaneously.

We have demonstrated that HBK001 can inhibit DPP4 enzymatic activity *in vitro* although the inhibitory potency is weaker than linagliptin. DPP4 activity *in vivo* is inhibited up to 50% by HBK001 treatment (30 mg/kg), which can equally be achieved by 0.3 mg/kg of linagliptin treatment as previously reported^24^. This is not surprising as we designed HBK001 by sacrificing some DPP4 pharmacophore in order to add the GPR119 pharmacophore. In addition, HBK001 has no effect on either DPP8 or DPP9 activity, indicating that HBK001 is a selective DPP4 inhibitor similar to linagliptin^21, 24, 29^.

Besides GPR119, GPR40, GLP1R and GIP are also involved in glucose-stimulated insulin secretion (GSIS) in pancreatic β-cells^22, 30, 31^. We have shown that HBK001 can specifically activate GPR119 but not GPR40, GLP1R nor GIPR, therefore directly promote GSIS *ex* and *in vivo* through GPR119-dependent signaling. Intriguingly, we noticed that although the effect of HBK001 on GPR119 transactivation is less than APD597, the insulin secretion in primary islets, as well as Ins1/2 transcription, was more robustly induced by HBK001 than by APD597 at the same concentration. Nevertheless, the detail underlying mechanism of HBK001-induced insulin secretion and gene transcription requires further investigation.

Consistent with previous studies^14, 32^, our data supports the argument that combining a DPP4 inhibitor and a GPR119 agonist treatment is much more efficient than a single drug alone. Firstly, HBK001 significantly improves blood incretins levels in ICR mice while linagliptin does not. Secondly, linagliptin fails to ameliorate hyperglycemia in diabetic KKAy mice despite the fact that serum DPP4 was over 95% inhibited, while HBK001 can effectively regulate glycemic control although DPP4 inhibition is only up to 50%. Thirdly, HBK001 can attenuate hyperglycemia and improve insulin resistance via increasing endogenous GLP-1 levels and directly stimulating insulin secretion, all of which cannot be achieved by linagliptin treatment. Overall, HBK001 could potentially provide a new therapeutic choice for T2DM patients who are insensitive to current DPP4 inhibitory treatment.

How does HBK001 improve β-cell function? Ansarullah *et al*.^14^ have previously shown that a combination of a GPR119 agonist and a DPP4 inhibitor stimulated β-cell replication and increased β-cell mass. We have confirmed that long-term treatment of HBK001 can significantly rescue the abnormal distribution of β- and α-cells and markedly increase β-cell percentage, therefore improving β-cell function, ameliorating OGTT as well as enhancing first-phase insulin secretion. The upregulation of pancreatic β-cell mass can be induced by β-cell regeneration (neogenesis and replication). Our data suggests that different transcription factors involved in β-cell function, such as NeuroD, Nkx6.1, Nkx2.2 and MafA^33–36^, are up-regulated by HBK001 treatment, which is consistent with other studies using GPR119 agonists^27, 31^ and DPP4 inhibitors^37^.

Taken together, for the first time, we have demonstrated that HBK001, a novel dual-target compound for GPR119 and DPP4, significantly improved glucose homeostasis and β-cell function by enhancing plasma GLP-1 levels and insulin secretion in β-cells, and therefore represents a very promising therapeutic candidate for diabetes treatment.

## Materials and Methods

### Chemicals and antibodies

HBK001 and UAMC00132 ((2S, 3R)-2-(2-amino-3-methyl-1-oxopentan-1-yl)-1, 3-dihydro-2H-isoindole hydrochloride, a DPP8/9 selective inhibitor) were synthesized in-house^38, 39^. The DPP4 inhibitor, linagliptin, GPR119 agonist, APD597, GPR40 antagonist, GW1100 and GLP-1 receptor (GLP1R) agonist, Exendin-4, were purchased from MedChem Express (USA). The GPR40 agonist, GW9508, was purchased from Cayman (USA). The GIPR agonist, GIP peptide (1-39), was purchased from TOCRIS Bioscience (USA). The GLP1R antagonist, Exendin fragment 9-39 (Ex 9-39), the synthetic substrate of DPP enzyme, Gly-Pro-*p*-nitroanilide, the Adenylyl Cyclase inhibitor, MDL12330A, and the PKA inhibitor, H-89, were purchased from Sigma Aldrich (USA). The DPP4 enzyme was purchased from Sino Biological Inc. (China). Lipofectamine 2000 was obtained from Invitrogen (USA). Antibodies, including anti-insulin, anti-glucagon (R&D Inc., USA), anti-PCNA, anti-CREB and anti-phosho-CREB (Cell Signaling Technology Inc., USA) were used in Western blot assays and immunostainings.

### Cells

HEK293 cells and mouse pancreatic *β*-cell line NIT-1 cells were obtained and cultured as previously described^21, 40^. Rat insulinoma INS-1 cells were obtained from the Cell Resource Center, Peking Union Medical College (which is the headquarters of National Infrastructure of Cell Line Resource, NSTI) and were cultured in RPMI-1640 medium (Gibco) containing 11 mM glucose and supplemented with 10 mM HEPES, 10% fetal bovine serum (Gibco, US), 2 mM L-glutamine, 1 mM sodium pyruvate, and 50 μM β-mercaptoanol. The cell line was checked free of mycoplasma contamination by PCR and culture. Its species origin was confirmed with PCR. The identity of the cell line was authenticated with STR profiling (FBI, CODIS). All the results can be viewed on the website (http://cellresource.cn).

### Human Pancreatic Islet isolation

Human pancreata were obtained from organ procurement organizations and transported to the cell isolation facility, Tianjin First Center Hospital. The procedures of islet isolation were performed as previously described^41^. All the methods and protocols were carried out in accordance with guidelines and regulations by the Medical Ethics Committee of Tianjin First Center Hospital and informed consent was obtained from every individuals. All the experimental protocols including any relevant details were approved by the Medical Ethics Committee of Tianjin First Center Hospital. In general, the pancreata were distended with Serva NB1 enzymes (Serva, Germany) after trimming, then the tissue was digested using the modified Ricordi semi-automatic method for 8–10 minutes and washed and collected for further purification. After incubation in UW solution for 30 min, the digested tissue was purified using UIC-UB gradient^42^ in a Cobe 2991 cell separator (Cobe 2991, Cobe, USA). Finally, the high purity islets (more than 70%) were collected and cultured in CMRL culture media (Mediatech, USA) containing IGF (Cell Sciences, USA) and 20% human albumin (CSL Behring GmbH, Germany) at 37 °C in 5% CO2 incubator.

### Animals

Male ICR mice, female diabetic KKAy mice^37, 43, 44^ and db/db (BKS.Cg-m+/+ *Lepr*db/J) mice were obtained and maintained as previously described^44^. All animal experiments were carried out according to the Standards for Laboratory Animals (GB14925-2001) and the Guideline on the Humane Treatment of Laboratory Animals (MOST 2006a), and all animal procedures were approved by Beijing Administration Office of Laboratory Animal (approval number: SCXK-Beijing-2009-0004).

### Plasmids information

Some plasmids used in this research were established in our lab. Peak13-CD5L-hGPR119, Peak13-CD5L-hGLP1R, Peak13-CD5L-hGPR40 and Peak13-CD5L-hGIPR, were constructed by inserting cDNA fragments of human G protein-coupled receptor 119 (GPR119, full length, Gene ID 139760), human glucagon like peptide 1 receptor (GLP1R, full length, Gene ID 2740), human G protein-coupled receptor 40 (GPR40, full length, Gene ID 2864) and human gastric inhibitory polypeptide receptor (GIPR, full length, Gene ID 2696) into Peak13-CD5L vector; pcDNA3.1-Gal4-CREB was constructed by fusing galactose-responsive transcription factor 4 (Gal4, 1Met-147Ser, Gene ID 855828) with human cAMP responsive element binding protein 1 (CREB1, 1Met-283Ala, Gene ID 1385) into pcDNA3.1 vector (Invitrogen, USA). Peak12-Gal4UAS-luci or Peak12-RIPCRE-luci, which expresses a luciferase reporter gene triggered by upstream activator sequence (UAS) or cAMP responsive element in rat insulin promoter, was constructed by inserting 5′CGGAAGACTCTCCTCCG3′ or 5′CTAGTGTTGACGTCCAAGTTGACGTCCAAT3′ into Peak12-luci vector; pCMV-Gal4-hElk1 was constructed using the pCMV vector (Clontech, USA) to express fused protein of Gal4 (1Met-147Ser) and human ETS transcription factor (Elk1, Trp379-Pro428, Gene ID 2002).

Peak12-Ins1/2-luci was constructed by inserting 1100 bp (−1000 to +100 bp) upstream sequence of mouse insulin gene 1/2 (Ins1/2, Gene ID 16333 and 16334) into Peak12-luci vector to construct a luciferase reporter triggered by the promoter of Ins1/2.

### Transactivation assay of GPR119, GLP1 receptor (GLP1R), GPR40 and GIPR

To perform a GPR119 transactivation assay, plasmids including Peak13-CD5L-hGPR119, pcDNA3.1-Gal4-CREB, Peak12-Gal4UAS-luci and pRL-TK (Promega, USA) were transiently co-transfected into HEK293 cells using Lipofectamine 2000. After 24 hours of transfection, the cells were transferred into 96-well plates and treated with the designated concentrations of compounds for another 24 hours, while using DMSO as a vehicle control. Then luciferase activity was measured by firefly and renilla luciferase assay kit (Vigorous Biotechnology, China). The activity fold change was determined as chemiluminescene value of compound treatment versus vehicle treatment.

The GLP1R transactivation assay was established in our lab and reported previously^45–47^. Briefly, NIT-1 cells were transfected with Peak12-RIPCRE-luci and then incubated with compounds. Luciferase activity was again used as the readout of the assay.

The GPR40 transactivation assay was performed by co-transfecting HEK293 cells with Peak13-CD5L-hGPR40, pCMV-Gal4-hElk1, Peak12-Gal4UAS-luci, and pRL-TK, followed by the same procedures as described above for the GPR119 transactivation assay.

The GIPR transactivation assay was performed according to the procedures for the GPR119 transactivation assay but replaced Peak13-CD5L-hGPR119 with Peak13-CD5L-hGIPR when performing the transfection.

### Evaluate the compounds’ effects on mouse insulin gene 1 and 2 (Ins1 and Ins2) transactivation

Luciferase reporter construction for the promoter of mouse insulin gene 1 and 2 (Ins1 and Ins2) and transcriptional activity assay were performed as previously described^48^. Briefly, Peak13-CD5L-hGPR119, Peak12-Ins1/2-luci and pRL-TK were co-transfected in HEK293 cells. After compound treatment, luciferase activity assay and the activity fold change were determined as described above.

### Single administration of HBK001 in ICR mice

To examine the single-dose effect of HBK001 on serum DPP4 activity, HBK001 (5 mg/kg and 30 mg/kg), linagliptin (2 mg/kg) or vehicle (distilled water) was orally administered to 4 h-fasted ICR mice. The dosage of HBK001 and linagliptin was determined according to previous studies^24, 29^ as well as their IC50 value of DPP4 inhibition *in vitro*. Blood samples were collected at 0, 1, 2, 4, 6 and 8 h after administration in order to measure serum DPP4 activity using a fluorometric DPP4 Activity Assay Kit (Sigma-Aldrich, cat. MAK088, USA). DPP4 activity was presented as the percentage of baseline level.

To examine the singe administration effect of HBK001 on total serum GLP-1 levels, HBK001 (30 mg/kg), APD597 (20 mg/kg), linagliptin (2 mg/kg) or vehicle (distilled water) was orally administered to 4 h-fasted ICR mice and 1 hour prior to the oral glucose load (2 g/kg). After 10 minutes of oral glucose load, blood samples of ICR mice were collected and total GLP-1 levels and GIP levels were measured using the GLP-1 Total ELISA Kit and GIP ELISA kit (Millipore, USA). In addition, blood acetaminophen levels were measured to explore gastric emptying of ICR mice after single administration of HBK001 (20 mg/kg) or APD597 (10 mg/kg) as previously described^49, 50^.

To analyze the acute effect of HBK001 on glucose excursion *in vivo*, the oral glucose tolerance test (OGTT) was performed in ICR mice. The animals were divided randomly into six groups: vehicle group (distilled water), different dosages of HBK001-treated groups (5, 10, 20, 30 mg/kg and the linagliptin-treated (2 mg/kg) group. HBK001 and linagliptin were orally administered to overnight-fasted mice 45 min prior to the oral glucose load (2 g/kg). Blood samples were collected from the tail tip before the glucose load (0 min) and at 30, 60, 120 min after the glucose load to measure blood glucose levels.

### Glucose-stimulated insulin secretion (GSIS) and intracellular cAMP production determination

Rat insulinoma INS-1 cells were prepared in a 96-well plate prior to GSIS determination. Primary islets were isolated from ICR mice, KKAy mice and human donor and GSIS determination was performed as previously described^51^. Insulin ELISA kit for rat and mouse (Alpco, USA) and human (Mercodia, Sweden) were used individually to determine insulin level. For intracellular cAMP production determination, NIT-1 cells were used and followed the same procedure as GSIS determination. After incubation with indicated compounds, the cell lysates was obtained by repeated freezing and thawing and collected to measure intracellular cAMP levels using a cAMP ELISA kit (NJJCBIO, China).

### Long-term treatment with HBK001 in diabetic db/db mice and KKAy mice

To compare the glucose-lowering effect of HBK001 and linagliptin in a diabetic animal model, db/db mice ranging from 35-45 g in weight with fasting blood glucose levels >180 mg/dl, were selected for the experiments. Mice were randomly divided into the following three groups: vehicle group (distilled water), HBK001 group (30 mg/kg per day) and linagliptin group (2 mg/kg per day). Vehicle and compounds were administrated by gavage every day for 5 weeks. During the long-term treatment, fasting blood glucose levels were monitored every week. The OGTT assay was performed on the 28^th^ day in a standard manner.

In a parallel experiment, KKAy mice, ranging from 45–55 g with fasting blood glucose levels >180 mg/dl, were selected and randomly divided into the same three groups as mentioned above. Vehicle and compounds were given orally every day for 6 weeks. Similarly, fasting blood glucose levels were monitored every week. OGTT assay was performed on the 33^rd^ day. At the end of the treatment, half of the mice were used for hyperglycemic clamp testing as previously described^52^, while the other half were decapitated for sample collections. Plasma samples were collected for insulin and glucagon level measurements (R&D Inc., USA). The pancreas was immediately removed and fixed in Bouin’s solution to perform immunofluorescence analysis as previously described^40^ or frozen for RNA isolation and subsequent qRT-PCR analysis.

### Quantitative Real-time PCR

Total RNA was extracted from frozen pancreas using TRIzol reagent (Invitrogen, USA). The cDNA was synthesized and quantitative real-time PCR was performed as previously described^44^. Results were expressed as fold expression relative to expression in the vehicle control group using the delta-delta Ct (^ΔΔ^Ct) method. The level of *β*-actin was used as an internal standard.

Primer sequences used were as follows:

NeuroD, forward 5′-CTGCAAAGGTTTGTCCC-AGC-3′,

reverse 5′-GGGGACTGGTAGGAGTAGGG-3′;

Nkx6.1, forward 5′-CCGCGCCTCCC-AACCTTGTT-3′,

reverse 5′-TTCTCCACCCCCGCGGGAAA-3′;

Nkx2.2, forward 5′-CCCATA-GTGTCCGCAAGGTG-3′,

reverse 5′-ACACTCCAAGGGACAAGCAC-3′;

MafA, forward 5′-GCGCCTCAGGAAAAGCGGTG-3′,

reverse 5′-AGCGCCTCGGGGTTCAGGTG-3′;

Ins1, forward 5′-GAAGCTTGTGATAAAACACCAGGA-3′,

reverse 5′-TGGCATTTACACGGTTGCCT-3′;

Ins2, forward 5′-CCATCAGCAAGCAGGAAGGTTA-3′,

reverse 5′-GCTTGACAAAAGCCTGG-GTG-3′.

β-actin, forward 5′-ACTCTTCCAGCCTTCCTTC-3′,

reverse 5′-ATCTCCTTCTGCATCCTGTC-3′.

### Data analysis

All values are presented as mean ± SEM. Data was analyzed by one-way ANOVA with Bonferroni’s correction and Student’s *t*-test. The value of *P* < 0.05 was accepted as being statistically significant.

## Electronic supplementary material

Supplementary figures and legends
