# Supplementary material for: The dual DPP4 inhibitor and GPR119 agonist HBK001 regulates glycemic control and beta cell function ex and in vivo
Source: Sci Rep. 2017 Jun 28;7:4351. doi: 10.1038/s41598-017-04633-5 (PMC5489512; doi:10.1038/s41598-017-04633-5)
Supplement: Supplementary file 1 — Supplementary figures and legends [file 41598_2017_4633_MOESM1_ESM.pdf]

The dual DPP4 inhibitor and GPR119 agonist HBK001 regulates glycemic control and beta cell function *ex* and *in vivo*

Yi Huan[1,3], Qian Jiang[1,3], Gang Li[1], Guoliang Bai[1], Tian Zhou[1], Shuainan Liu[1], Caina Li[1], Quan Liu[1], Sujuan Sun[1], Miaomiao Yang[1], Nan Guo[1], Xing Wang[1], Shusen Wang[2], Yaojuan Liu[2], Guanqiao Wang[2], Haihong Huang[1]*, Zhufang Shen[1]*

1. State Key Laboratory of Bioactive Substances and Functions of Natural Medicines, Institute of Materia Medica, Chinese Academy of Medical Sciences and Peking Union Medical College, Beijing, China.
2. Organ Transplant Center, Tianjin First Center Hospital, Tianjin, China
   Key Laboratory for Critical Care Medicine of the Ministry of Health, Tianjin First Center Hospital, Tianjin, China
3. These authors contribute equally.

*Correspondence to Haihong Huang (joyce@imm.ac.cn) and Zhufang Shen (shenzhf@imm.ac.cn).

SUPPLEMENTARY FIGURE LEGENDS

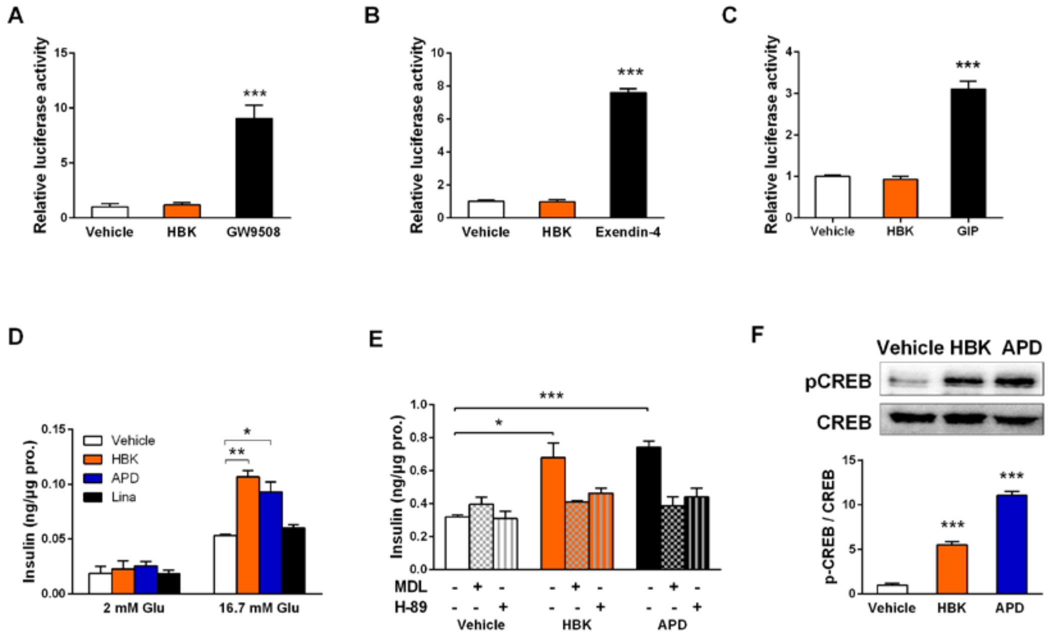

Supplementary Figure 1. HBK001 selectively activates GPR119 and promotes glucose-stimulated insulin secretion (GSIS). (A) Effect of HBK001 on GPR40. Peak13-CD5L-hGPR40, pCMV-Gal4-hElk1, Peak12-Gal4UAS-luci were transiently transfected in HEK293 cells. GW9508, an agonist of GPR40, was used as a positive control. (B) Effect of HBK001 on GLP1R. Peak12-RIPCRE-luciferase was transiently transfected in NIT-1 cells. Exendin-4 (1 μM), an agonist of GLP1R, was used as a positive control. (C) Effect of HBK001 on GIPR. Peak13-CD5L-hGIPR, pcDNA3.1-Gal4-CREB and Peak12-Gal4UAS-luci plasmids was transiently transfected in HEK293 cells. GIP peptide (1-39) was used as a positive control at the concentration of 1 μM. (D) Insulin secretion in INS-1 cells with exposure to indicated concentrations of glucose and different compounds. DMSO was used as the vehicle control. (E) Glucose-stimulated insulin secretion (GSIS) determination in primary

islets of ICR mice. Primary islets isolated from ICR mice were exposed to 16.7 mM glucose and HBK001 or APD597 while solvent DMSO was used as a vehicle control. Meanwhile, the inhibitor of adenylyl cyclase, MDL12330A (MDL), or protein kinase A, H-89, was added to block the production of cyclic adenosine monophosphate (cAMP) and the following cascade activation. (F) Western blot analysis of protein levels of the phosphorylated CREB in NIT-1 cells treated with HBK001 and the GPR119 agonist, APD597. Solvent DMSO was used as the vehicle control. In all of the above experiments, the concentration of compounds was 10 μM or specially indicated. Data are presented as mean ± SEM (n = 3), * $P < 0.05$, ** $P < 0.01$, *** $P < 0.001$ versus Vehicle.

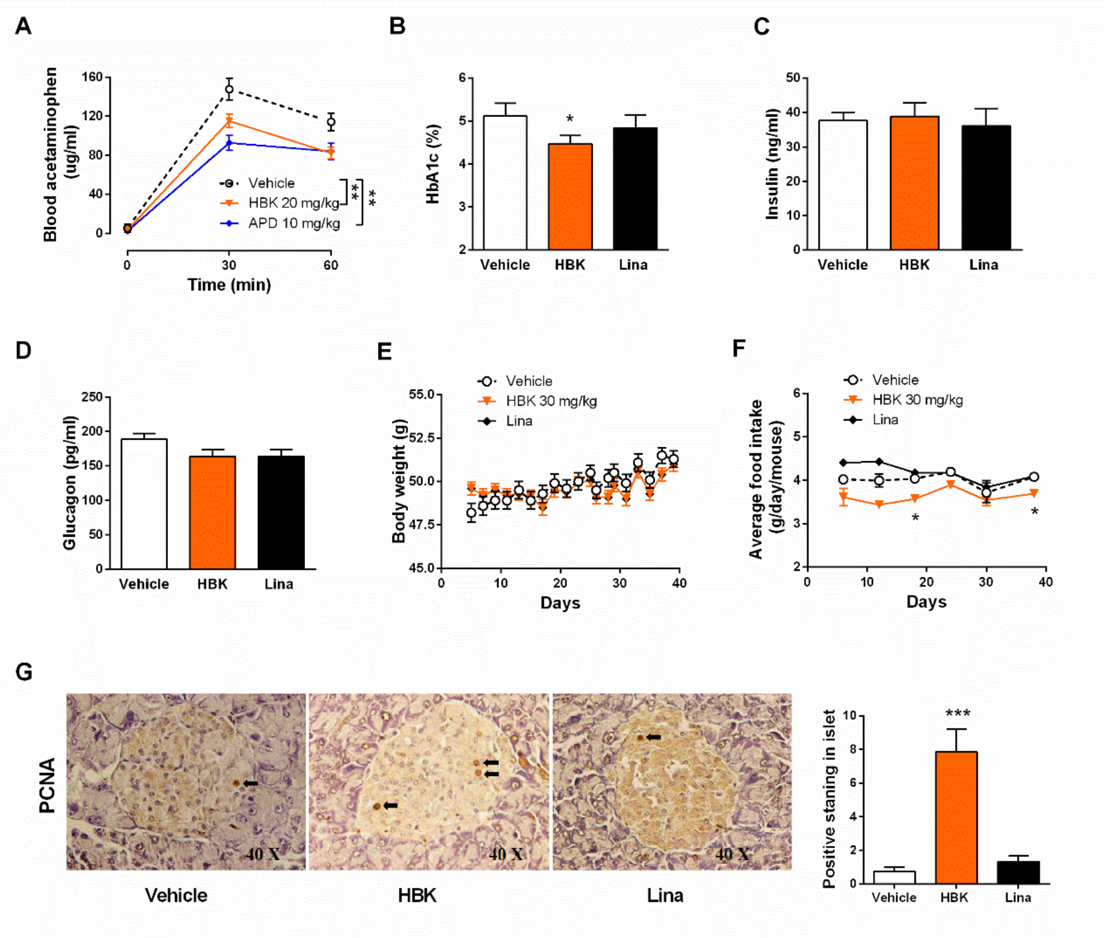

Supplementary Figure 2. HBK001 ameliorated hyperglycemia in vivo with mild inhibition of food intake and gastric emptying. (A) Gastric emptying effect of HBK001 or APD597 in ICR mice was determined by measurement of blood acetaminophen levels 30 min and 60 min after gavage of acetaminophen. ICR mice were orally administrated vehicle (distilled water), HBK001 (20 mg/kg) or APD597 (10mg/kg). (B) HbAlc levels were measured on the 41st day of the treatment. (C) Fasting insulin levels analyzed on the 48th day of treatment. (D) Fasting glucagon levels analyzed on the 48th day of treatment. KKAy mice were orally administrated vehicle (distilled water), HBK001 (30 mg/kg) or linagliptin (2 mg/kg) for 5 weeks and the above determinations were performed. (E-F) Body weight (E) and food intake (F) of KKAy mice orally administrated with vehicle (distilled water), HBK001 (30 mg/kg)

or linagliptin (2 mg/kg) were monitored every two days for 5 weeks. (G) HBK001 promoted cell proliferation in islets of KKAy mice. Immunostaining of PCNA in pancreatic section from KKAy mice after 5-week of oral administration with vehicle (distilled water), HBK001 (30 mg/kg) or linagliptin (2 mg/kg). Data are presented as mean ± SEM (A-F, n = 10; G, n=5), * $P < 0.05$, *** $P < 0.001$ versus Vehicle.
